# Myocardial Injury as a Prognostic Factor in Mid- and Long-Term Follow-Up of COVID-19 Survivors

**DOI:** 10.3390/jcm10245900

**Published:** 2021-12-16

**Authors:** Andrea Izquierdo, Diana Mojón, Alfredo Bardají, Anna Carrasquer, Alicia Calvo-Fernández, José Carreras-Mora, Teresa Giralt, Sílvia Pérez-Fernández, Núria Farré, Cristina Soler, Clàudia Solà-Richarte, Paula Cabero, Beatriz Vaquerizo, Jaume Marrugat, Núria Ribas

**Affiliations:** 1 Cardiology Department, Hospital del Mar, Passeig Marítim de la Barceloneta 25-29, 08003 Barcelona, Spain; dmojon@psmar.cat (D.M.); acalvofernandez@psmar.cat (A.C.-F.); jcarrerasmora@psmar.cat (J.C.-M.); tgiralt@psmar.cat (T.G.); nfarrelopez@psmar.cat (N.F.); csoler@imim.es (C.S.); csola@psmar.cat (C.S.-R.); pcabero@imim.es (P.C.); beavaquerizo@yahoo.es (B.V.); nribasbarquet@psmar.cat (N.R.); 2 Medicine Department, Autonomous University of Barcelona, Bellaterra, 08193 Barcelona, Spain; 3 Cardiology Department, Joan XXIII University Hospital of Tarragona, 430005 Tarragona, Spain; abardaji.hj23.ics@gencat.cat (A.B.); carrasquer1987@gmail.com (A.C.); 4 Institut Investigació Sanitària Pere i Virgili (IISPV), 43007 Tarragona, Spain; 5 Medicine Department, Rovira i Virgili University, 43002 Tarragona, Spain; 6 Medicine Department, Pompeu Fabra University, 08005 Barcelona, Spain; 7 Scientific Coordination Facility, Biocruces Bizkaia Health Research Institute, 48903 Barakaldo, Spain; silvia_medina2712@hotmail.com; 8 Heart Disease Biomedical Research Group, IMIM (Hospital del Mar Medical Research Institute), 08003 Barcelona, Spain; 9 CIBER Group in Epidemiology and Public Heath (CIBERESP), Hospital del Mar Medical Research Institute (IMIM), 08003 Barcelona, Spain; jaume@imim.es; 10 REGICOR (Registre Gironí del Cor) Study Group, IMIM (Hospital del Mar Medical Research Institute), 08003 Barcelona, Spain

**Keywords:** myocardial injury, COVID-19, long-term, prognosis, mortality, readmission

## Abstract

Myocardial injury, which is present in >20% of patients hospitalized for COVID-19, is associated with increased short-term mortality, but little is known about its mid- and long-term consequences. We evaluated the association between myocardial injury with one-year mortality and readmission in 172 COVID-19 patients discharged alive. Patients were grouped according to the presence or absence of myocardial injury (defined by hs-cTn levels) on admission and matched by age and sex. We report mortality and hospital readmission at one year after admission in all patients and echocardiographic, laboratory and clinical data at six months in a subset of 86 patients. Patients with myocardial injury had a higher prevalence of hypertension (73.3% vs. 50.0%, *p* = 0.003), chronic kidney disease (10.5% vs. 2.35%, *p* = 0.06) and chronic heart failure (9.3% vs. 1.16%, *p* = 0.03) on admission. They also had higher mortality or hospital readmissions at one year (11.6% vs. 1.16%, *p* = 0.01). Additionally, echocardiograms showed thicker walls in these patients (10 mm vs. 8 mm, *p* = 0.002) but without functional disorder. Myocardial injury in COVID-19 survivors is associated with poor clinical prognosis at one year, independent of age and sex, but not with echocardiographic functional abnormalities at six months.

## 1. Introduction

Severe acute respiratory syndrome coronavirus 2 (SARS-CoV-2) has brought about the COVID-19 pandemic, which has caused significant mortality and morbidity worldwide. The symptoms of COVID-19 can range from none or mild to a broad spectrum of clinical features, including severe respiratory failure and myocardial injury, and can lead to death [1]. The epidemiological and clinical characteristics, pathogenesis, and complications of patients with COVID-19 during the acute phase have been described [2,3], but the long-term consequences of the disease are still unclear. Only a few studies with a limited sample size have followed up their patients after discharge [4,5,6,7,8,9], and the patient characteristics determining mid- and long-term prognosis remain largely unknown.

As previously reported by our group and others, more than 20% of patients hospitalized with COVID-19 suffer myocardial injury, defined by elevated high-sensitivity cardiac troponin (hs-cTn) levels [10,11,12,13,14,15]. Acute respiratory infections and sepsis often entail elevated serum cardiac markers, which are in turn associated with higher short- and mid-term mortality, even after recovery from infection [16,17,18,19]. While myocardial injury has been associated with worse in-hospital outcomes in COVID-19 patients [10,11,12,13,14,20,21,22,23,24,25], data on mid- and long-term prognosis are scarce [4,5,7,26,27,28]. Since age and sex are significant in-hospital prognostic factors [21,22] for COVID-19 patients with myocardial injury, we hypothesized that these factors might also play a role in mid- and long-term prognosis and could be potential confounding variables if not controlled for.

In the present study, patients were classified in two groups according to the presence or absence of myocardial injury during the acute phase of COVID-19 infection and matched for age and sex in an attempt to better understand the effect of myocardial injury on mid- and long-term prognosis, while eliminating the potential confounding effect of age and sex. We report mortality and readmission outcomes in all patients at one year after initial hospitalization and details on echocardiographic, laboratory and clinical characteristics in a subgroup of patients at six months after hospitalization.

## 2. Methods

We designed a two-centre cohort study of 172 survivors of COVID-19 after hospitalization. All 172 patients were classified in two groups according to the presence or absence of myocardial injury during the acute phase of COVID-19 infection and matched for age and sex. We analysed mortality and the need for hospital readmission during the first year after initial admission. A subgroup of 86 patients, also matched for age and sex, were re-examined through interviews, echocardiograms and laboratory tests six months after initial admission.

**Figure 1:**
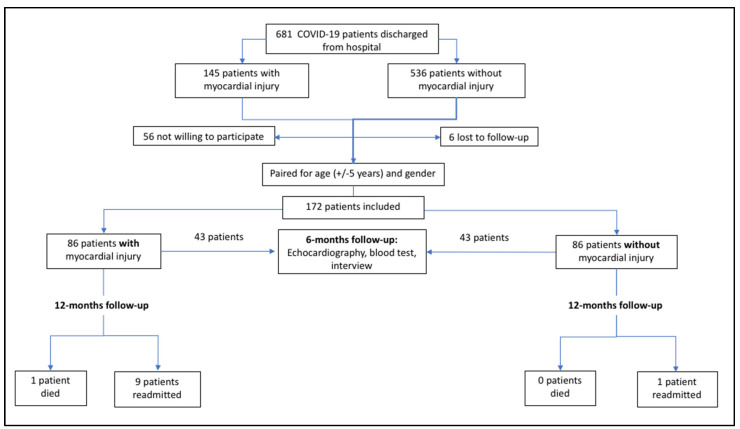
Flow chart showing inclusion of patients with COVID-19 in the study.

We selected a consecutive series of patients ≥18 years of age with laboratory-confirmed COVID-19 who were admitted to one of two Spanish hospitals (Hospital del Mar, Barcelona, and Joan XXIII University Hospital of Tarragona, Tarragona, Spain) from 27 February to 16 May 2021 and subsequently discharged alive. Of a total of 681 eligible patients, 145 patients had myocardial injury during the acute phase and 86 of these patients with myocardial injury could be paired 1:1 for age (+/−5 years) and sex with another 86 patients without myocardial injury. Patients were excluded if they were unwilling to participate, were lost to follow-up, or could not be matched 1:1 by age and sex (Figure 1).

The acute phase of infection was defined as the time between hospital admission and hospital discharge. Demographic and clinical data were retrieved from electronic medical records. Demographic data included age, sex and smoking history. Clinical data included cardiovascular risk factors, defined as previous history or current treatment for hypertension, dyslipidaemia or diabetes mellitus in the patient’s medical records; chronic kidney disease, defined as a persistent abnormality in kidney function (glomerular filtration rate <60 mL/min/1.73 m^2^); and previous history or current treatment in the patient’s medical records for chronic heart failure, coronary heart disease, atrial fibrillation, chronic obstructive pulmonary disease, cerebrovascular disease, or peripheral vascular disease.

Diagnosis of COVID-19 was performed either by RT-PCR on a nasopharyngeal swab test of the upper respiratory tract or antigen test for SARS-CoV2. Both the RT-PCR assays and the antigen tests were performed in accordance with World Health Organization (WHO) guidelines [1]. Laboratory analyses at admission and at follow-up included blood count, kidney function, C-reactive protein, lactate dehydrogenase, D-dimer, NT-proBNP and hs-cTn. In accordance with the Fourth Universal Definition of Myocardial Infarction [29], myocardial injury was diagnosed if serum levels of hs-cTn within 48 h of admission were above the 99th percentile upper reference limit (hs-cTn-T > 14 ng/L and hs-cTn-I > 47 ng/L). At Hospital del Mar, hs-cTn-T plasma levels were measured using an electrochemiluminescence-based immunoanalytical system (Elecsys 2010, Roche Diagnostics Ltd., Mannheim, Germany), and at Joan XXIII University Hospital of Tarragona, hs-cTn-I concentrations were measured with an automated immunoassay (High-Sensitivity troponin I Assay, Advia Centaur, Siemens Healthineers, Erlangen, Germany).

A subset of 86 patients (43 with and 43 without myocardial injury, matched for age and sex) had a follow-up visit in the outpatient clinic six months after hospital discharge. All patients were interviewed with questionnaires on symptoms and employment situation, and the same laboratory analyses were performed as at admission. A standard echocardiogram was performed using CX 50 (Philips Medical Systems, Bothell, WA, USA) by senior expert cardiologists. Left ventricular diameters, interventricular and posterior wall thickness were measured with M Mode. Left ventricular ejection fraction (LVEF) was measured with the biplane Simpson’s method [30]. Measurements of mitral inflow included the peak early filling (E-wave) and late diastolic filling (A-wave) velocities. Early diastolic mitral septal and lateral annular velocities (e′) were measured in the apical 4-chamber view [31]. E/e’ ratio and systolic pulmonary artery pressure (PAP) were calculated. Right ventricular (RV) function was evaluated by tricuspid annular plane systolic excursion (TAPSE).

All 172 patients were followed up at one year after initial hospital admission by telephone contact and through electronic medical records. Hospital readmission was defined as a subsequent hospital stay longer than 24 h. Cardiovascular causes of admission were defined as those related to arrhythmia, a cardiac ischemic event or heart failure.

The primary outcomes were calculated for all patients at one year from initial hospital admission for COVID-19 and included death and/or hospital readmission due to any cause. The two primary outcomes were calculated both separately and as a composite outcome. Secondary outcomes were calculated for the subset of 86 patients at six months from initial hospital admission and included echocardiographic, laboratory and clinical characteristics.

Continuous variables were described as mean (±standard deviation [SD]) or median (interquartile range [IQR]) according to their distribution. Normal distribution was assessed with the Shapiro–Wilk test and normal Q-Q plot. Categorical variables were presented as percentages. Differences between groups were assessed using the Student’s T-test or Mann–Whitney U test, as appropriate, for continuous variables and the Chi-square test for categorical variables. Curves for deaths and hospital readmissions were generated by the Kaplan–Meier method and compared with the log-rank test. All statistical analyses were performed with R version 3.6.1 (R Foundation for Statistical Computing, Vienna, Austria). Statistical significance was set at *p* ≤ 0.05.

## 3. Results

### 3.1. Patients

Of 681 eligible patients, a total of 172 patients paired for age and sex (86 with and 86 without myocardial injury) were followed up during a period of 358 days (IQR 350–374) after their initial hospital admission for COVID-19. Table 1 displays the baseline patient characteristics and results of laboratory analyses for all patients according to the presence or absence of myocardial injury. Patients with myocardial injury had a higher prevalence of hypertension, chronic kidney disease and chronic heart failure than those without myocardial injury.

### 3.2. Myocardial Injury and Long-Term Prognosis

**Figure 2:**
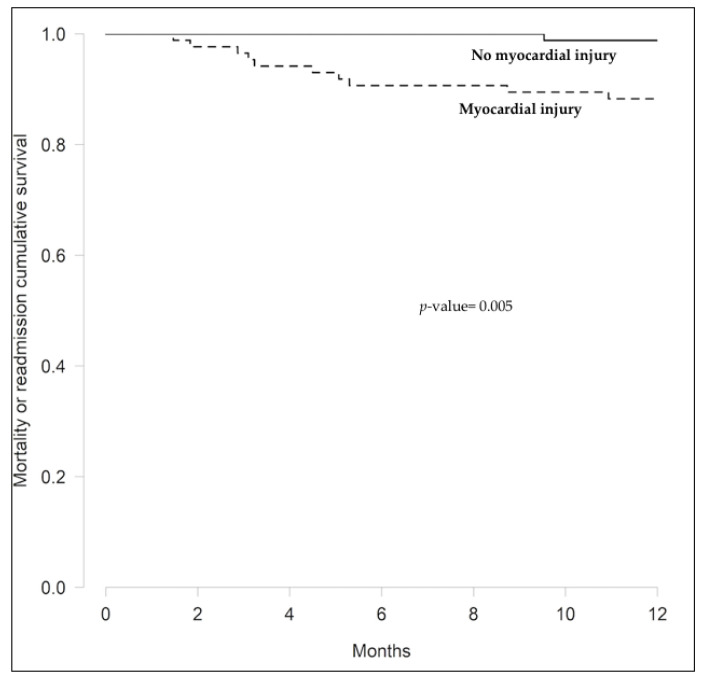
Kaplan–Meier curves for mortality and/or hospital readmission in 172 COVID-19 survivors according to the presence or absence of myocardial injury on initial hospital admission.

At one year after initial admission, one patient with myocardial injury had died due to an unknown cause. Hospital readmission was higher among patients with myocardial injury. The composite primary endpoint (death or readmission) occurred more often in patients with myocardial injury (Table 2 and Figure 2). In addition, time between discharge and hospital readmission was shorter in patients with myocardial injury (173 ± 123 vs. 458 ± 142 days; *p* = 0.006).

### 3.3. Echocardiographic, Laboratory and Clinical Characteristics at Six Months

The subset of 86 patients (43 with and 43 without myocardial injury at initial admission) were re-examined at six months after initial admission (median follow-up 229.5 days [IQR 174.5–252.75]). There were no relevant differences in baseline characteristics between these 86 patients and the other 86 patients in the study who were not re-examined at six months (Supplementary Table S1).

Table 3 shows the characteristics of the 86 patients at 6-month follow-up. Echocardiograms revealed that the 43 patients with myocardial injury had significantly thicker walls (interventricular septum and posterior wall) and PAPs than those without myocardial injury. However, there were no significant differences in biventricular function or wall motion abnormalities (2.8% in both groups) and no clinically relevant functional disorder was detected. The mean hs-cTn level for all 86 patients decreased from 17.2 to 7.13 ng/dL (*p* < 0.001) between initial admission and 6-month follow-up, while approximately half of the patients (48.8%) with myocardial injury showed persistent elevated hs-cTn levels at 6-month follow-up. Patients with myocardial injury also had worse kidney function, less haemoglobin, more lymphopenia and higher NT-proBNP levels at six months. Most of the 86 patients were classified as New York Health Association (NYHA) functional class I or II, and we found no differences in symptoms at six months according to myocardial injury status on admission. However, 11.6% of patients with myocardial injury remained on sick leave at 6 months post-discharge, compared to 2.33% of those without myocardial injury (*p* = 0.082).

## 4. Discussion

We have evaluated mid- and long-term outcomes in a cohort of COVID -19 survivors matched for age and sex. We found that patients with myocardial injury on hospital admission had poorer 1-year prognosis than those without myocardial injury, mainly in terms of hospital readmission. In addition, myocardial injury on admission was related to thicker myocardial walls and higher PAPs on echocardiograms performed six months after initial admission.

Myocardial injury, an important pathogenic feature of COVID-19, is associated with elevated in-hospital mortality [10,11,12,13,14,20,21,22,23,24,25]. In our cohort, 21% of all patients had myocardial injury on admission, which is in line with findings in other studies [12,20,21]. SARS-CoV2 infection can affect the heart through various mechanisms, ranging from direct viral damage mediated by the angiotensin-converting enzyme-2 receptor expressed on cardiomyocytes to indirect damage secondary to the systemic inflammatory response [32,33,34,35]. Indeed, systemic infections such as viral pneumonia not only increase the imbalance of oxygen supply and demand to the heart [36,37] but also yield inflammatory factors that trigger atherosclerotic plaque instability [32,33]. Recent autopsy-based studies identified fibrin-rich microthrombi and microangiopathy as the main causes of myocardial necrosis associated with COVID-19 [38,39]. In contrast, in a recent study with cardiovascular magnetic resonance, the most prevalent pattern of myocardial injury was myocarditis-like (27%), followed by ischemia-like (22%), and dual pathology (6%), with no evidence of fibrosis during the convalescent phase after severe COVID-19 [8].

There is evidence of increased short-term mortality associated with myocardial injury, but little is known about its mid- and long-term consequences [40,41,42]. In our cohort, patients with myocardial injury had a significantly higher incidence of 1-year mortality or hospital readmission than those without myocardial injury (11.6 vs. 1.16%, *p* = 0.013). In a previous study that included in-hospital events, Kini et al. also found that myocardial injury was a strong and independent predictor of 6-month mortality [9]. Along the same lines, in our cohort, patients with myocardial injury had a worse baseline profile (higher prevalence of hypertension, chronic kidney disease, and chronic heart failure), suggesting that the presence of myocardial injury may help to identify a subset of COVID-19 patients with a high risk of poor mid- and long-term prognosis.

At six months after initial hospital admission, nearly half of our patients with myocardial injury continued to have elevated serum levels of hs-cTn, potentially indicating chronic myocardial injury [29]. The presence of thicker myocardial walls in patients with myocardial injury may be explained, at least in part, by a higher prevalence of hypertension in these patients. The difference in PAPs between patients with and without myocardial injury was statistically significant but had no clinical relevance. An extremely low prevalence of wall motion abnormalities was found in both groups, with mostly preserved LVEF. Kotecha et al. obtained similar results with cardiac magnetic resonance in COVID-19 patients approximately two months after discharge [8]. Furthermore, most of our patients were in functional class NYHA I or II, which is in line with our echocardiographic findings.

A previous study by our group found that high hs-cTn levels predicted worse in-hospital prognosis in COVID-19 patients and that early measurement of troponin levels helped guide patient management during the acute phase of the disease [20]. Our results in the present study suggest that early hs-cTn measurement may also help to identify COVID-19 patients with a high risk of complications after discharge. Further investigation is warranted to confirm our findings and, particularly, to elucidate the pathological determinants of this association.

Our study has several limitations, including the fact that an echocardiogram is not the most sensitive or specific imaging technique to assess myocardial function, so it may not have detected subtle abnormalities that could have impacted future clinical prognosis. However, an echocardiogram is readily accessible and is the most frequently used imaging technique for assessing cardiac function in clinical practice. In addition, some patients did not agree to attend the planned follow-up at six months. However, baseline characteristics were similar in patients who were re-examined at six months and those who were not. Finally, due to the limited number of events in our study, we were not able to adjust the effect of myocardial injury for potential confounding factors. Although we paired patients by age and sex, thus eliminating these potential confounders, this resulted in a more limited number of patients, which, in turn, precluded adjusting for other potential confounders. We cannot rule out the possibility that comorbidities or COVID-19 severity (e.g., lymphopenia, increased D-Dimer or LDH) might have contributed to the worse one-year prognosis in the group of patients with myocardial injury [20]. Nevertheless, to the best of our knowledge, this study had one of the longest follow-up periods of COVID-19 survivors to date and our findings on the mid- and long-term prognosis of patients who present with myocardial injury can be useful in managing the follow-up of these patients.

## 5. Conclusions

The early presence of myocardial injury in hospitalized COVID-19 patients is associated with worse one-year prognosis, mainly in terms of a higher incidence of hospital readmissions. This association is independent of patient age and sex. However, myocardial injury is not associated with significant echocardiographic functional abnormalities at six months after initial hospital admission.

## Figures and Tables

**Table 1:** Baseline characteristics at hospital admission in COVID-19 patients with and without myocardial injury.

	Myocardial Injury (*n* = 86)	No Myocardial Injury (*n* = 86)	*p*-Value
Clinical characteristics			
Age, years *	70.5 (60–74)	69.0 (60–74)	0.692
Women	29.2%	31.95%	0.867
Hypertension	73.3%	50.0%	0.003
Dyslipidaemia	43.0%	32.6%	0.208
Ever smoked	29.6%	29.8%	1.00
Diabetes mellitus	30.2%	17.6%	0.080
Chronic kidney disease	10.5%	2.35%	0.064
Chronic heart failure	9.30%	1.16%	0.034
Coronary heart disease	10.5%	8.14%	0.793
Atrial fibrillation	12.8%	4.76%	0.115
COPD	11.6%	10.5%	1.00
Cerebrovascular disease	5.88%	2.33%	0.277
Peripheral vascular disease	4.65%	4.65%	1.00
Laboratory analysis results			
Leukocytes/µL×10 ^3^ *	7.63 (5.89–14.4)	5.95 (5.26–9.14)	0.115
Lymphocytes/µL×10^3^ *	0.98 (0.68–1.73)	1.25 (0.9–2.29)	0.039
Haemoglobin, g/dL **	13.1 (1.74)	13.9 (1.74)	0.008
Creatinine, mg/dL *	0.99 (0.79–1.3)	0.84 (0.68–1.02)	0.001
LDH U/L *	329 (270–449)	279 (240–356)	0.001
C-reactive protein, mg/dL *	10.0 (4.20–18.0)	7.10 (3.90–11.9)	0.020
D-Dimer, ng/mL *	950 (610–1920)	600 (420–1150)	0.002

* Median (inter-quartile range) ** Mean (standard deviation); COPD, chronic obstructive pulmonary disease; LDH, lactate dehydrogenase.

**Table 2:** Death and hospital readmission due to any cause at one year after hospital discharge.

	All Patients (*n* = 172)	Myocardial Injury (*n* = 86)	No Myocardial Injury (*n* = 86)	*p*-Value *
Composite endpoint **	11	10	1	0.01
Death	1	1	0	1.00
Hospital readmission				0.02
Total	10	9	1	
Cardiovascular cause	4	3	1	
Non-cardiovascular cause	6	6	0	

* *p*-value for comparison between patients with and without myocardial injury. ** Death or hospital readmission.

**Table 3:** Echocardiographic, laboratory and clinical characteristics of COVID-19 patients at six months after initial hospital admission.

	Myocardial Injury (*n* = 43)	No Myocardial Injury (*n* = 43)	*p*-Value
Echocardiographic characteristics			
Interventricular septum wall thickness, mm *	10.0 (9.00–12.0)	9.00 (8.00–10.5)	0.002
Posterior wall thickness, mm *	10.0 (9.00–11.0)	8.00 (7.00–10.0)	0.002
LV telediastolic diameter, mm *	50.0 (45.0–55.5)	49.0 (45.0–53.0)	0.659
LV telesistolic diameter, mm *	30.0 (26.5–34.5)	30.0 (27.0–32.5)	0.274
LV end diastolic volume, mL *	91.0 (81.0–108)	85.0 (75.5–101)	0.111
LV end systolic volume, mL *	33.0 (26.0–46.0)	32.0 (25.0–41.5)	0.380
LV ejection fraction (Simpson’s biplane), % *	63.0 (60.0–67.5)	63.0 (58.0–67.8)	0.833
TAPSE, mm *	23.0 (21.0–25.0)	23.0 (20.2–24.8)	0.951
E/E’ratio *	8.00 (6.2–9.50)	8.00 (6.00–9.05)	0.303
PAP, mmHg *	34.0 (27.5–37.8)	29.0 (27.0–31.0)	0.021
Laboratory characteristics			
Urea, mg/dL *	43.0 (35.0–52.0)	38.0 (32.5–44.6)	0.028
Creatinine, mg/dL *	1.01 (0.88–1.17)	0.91 (0.81–1.04)	0.041
Glomerular filtrate rate, mL/min/1.73 m^2^ **	71.5 (24.3)	82.9 (20.4)	0.021
C-Reactive Protein, mg/dL *	0.18 (0.10–0.37)	0.13 (0.08–0.24)	0.236
NT-proBNP, pg/L *	119 (73.9–234)	71.3 (32.9–120)	0.001
Haemoglobin, g/dL **	13.2 (1.40)	14.1 (1.29)	0.002
Leukocytes/µL×10^3^ *	7020 (6170–7790)	6600 (5395–7670)	0.260
Lymphocytes/µL×10^3^ *	1910 (1235–2205)	2080 (1710–2555)	0.062
D-Dimer, ng/mL *	295 (205–388)	230 (190–375)	0.137
Clinical characteristics			
Chest pain	4.65%	2.33%	1.00
Palpitations	4.65%	0.00%	0.494
NYHA functional class I-II NYHA functional class III-IV	93.0% 7.00%	97.7% 2.30%	0.407
Pulse oximetry *	98.0 (97.0–99.0)	97.0 (96.5–98.0)	0.098
Systolic blood pressure **	141 (17.0)	136 (18.6)	0.194
Diastolic blood pressure **	77.3 (11.7)	77.9 (13.2)	0.826

* Median (Inter-quartile range) ** Mean (standard deviation); LV, left ventricular; TAPSE, Tricuspid Annular Plane Systolic Excursion; PAP, pulmonary artery pressure; NT-proBNP, N-terminal prohormone of brain natriuretic peptide; NYHA, New York Heart Association.

## Supplementary Materials

The following are available online at https://www.mdpi.com/article/10.3390/jcm10245900/s1, Table S1: Baseline characteristics in patients re-examined and those not re-examined at six months.
