# Supplementary material for: Myocardial Injury as a Prognostic Factor in Mid- and Long-Term Follow-Up of COVID-19 Survivors
Source: J Clin Med. 2021 Dec 16;10(24):5900. doi: 10.3390/jcm10245900 (PMC8708676; doi:10.3390/jcm10245900)
Supplement: Supplementary file 1 [file jcm-10-05900-s001.zip › jcm-1505384-supplementary.pdf]

Table S1. Baseline characteristics in patients re-examined and those not re-examined at six months.

	Patients re-examined (n = 86)	Patients not re-examined (n = 86)	p-value
**Age, years***	69.0 (65.0–73.8)	67.0 (54.0–75.5)	0.387
**Women**	26.7%	36.4%	0.306
**Hypertension**	55.8%	67.4%	0.157
**Dyslipidemia**	39.5%	36.0%	0.753
**Ever smoked**	30.5%	28.9%	0.960
**Diabetes mellitus**	18.6%	29.4%	0.140
**Chronic kidney disease**	4.65%	8.24%	0.520
**Chronic heart failure**	3.49%	6.98%	0.496
**Coronary heart disease**	6.98%	11.6%	0.431
**Atrial fibrillation**	11.6%	5.95%	0.301
**COPD**	10.5%	11.6%	1.000
**Cerebrovascular disease**	3.53%	4.65%	1.000
**Peripheral vascular disease**	3.49%	5.81%	0.720
**Leukocytes/μLx10^3***	6.40 (5.25–8.2)	7.11 (5.55–8.85)	0.366
**Lymphocytes/μLx10^3***	0.90 (0.68–1.17)	1.08 (0.67–1.36)	0.201
**Hemoglobin, g/dL****	14.0 (1.54)	13.1 (1.88)	0.001
**Creatinine, mg/dL***	0.95 (0.78–1.16)	0.82 (0.69–1.02)	0.014
**LDH U/L***	316 (256–393)	291 (244–390)	0.352
**C-reactive protein, mg/dL***	8.70 (3.9–13.5)	8.75 (4.0–17.2)	0.411
**D-Dimer, ng/mL***	670 (460–1190)	880 (520–2050)	0.085

*Median (inter-quartile range); **Mean (standard deviation); COPD, Chronic Obstructive Pulmonary Disease; LDH, lactate dehydrogenase.
